# Three-dimensional printing of continuous-fiber composites by in-nozzle impregnation

**DOI:** 10.1038/srep23058

**Published:** 2016-03-11

**Authors:** Ryosuke Matsuzaki, Masahito Ueda, Masaki Namiki, Tae-Kun Jeong, Hirosuke Asahara, Keisuke Horiguchi, Taishi Nakamura, Akira Todoroki, Yoshiyasu Hirano

**Affiliations:** 1 Tokyo University of Science, 2641 Yamazaki, Noda, Chiba 278-8510, Japan; 2 Nihon University, 1-8-14 Kanda-surugadai, Chiyoda, Tokyo 101-8308, Japan; 3 Tokyo Institute of Technology, 2-12-1 O-okayama, Meguro, Tokyo 152-8551, Japan; 4 Japan Aerospace Exploration Agency, 6-13-1 Osawa, Mitaka, Tokyo 181-0015, Japan

## Abstract

We have developed a method for the three-dimensional (3D) printing of continuous fiber-reinforced thermoplastics based on fused-deposition modeling. The technique enables direct 3D fabrication without the use of molds and may become the standard next-generation composite fabrication methodology. A thermoplastic filament and continuous fibers were separately supplied to the 3D printer and the fibers were impregnated with the filament within the heated nozzle of the printer immediately before printing. Polylactic acid was used as the matrix while carbon fibers, or twisted yarns of natural jute fibers, were used as the reinforcements. The thermoplastics reinforced with unidirectional jute fibers were examples of plant-sourced composites; those reinforced with unidirectional carbon fiber showed mechanical properties superior to those of both the jute-reinforced and unreinforced thermoplastics. Continuous fiber reinforcement improved the tensile strength of the printed composites relative to the values shown by conventional 3D-printed polymer-based composites.

Three-dimensional (3D) printing^1^,^2^,^3^ or additive manufacturing^4^,^5^,^6^ enables the fabrication of near-net-shaped complex 3D parts without expensive molds or tools in short periods of time, based on 3D computer-aided design (CAD) data. 3D printing is expected to revolutionize the manufacturing of components. While several 3D printing systems are available^4^,^6^, printing based on fused-deposition modeling (FDM) using thermoplastics^7^,^8^,^9^ is particularly widespread because of the simplicity and potential applicability of the method. However, the mechanical properties of products fabricated by conventional FDM 3D printing are inherently poor because of the thermoplastic resins^10^,^11^ used, although the optimization of processing parameters, such as the lamination direction and laminate thickness, has been investigated for improving the mechanical properties of thermoplastic resin parts^12^. 3D printing has primarily been used for trial products or toys, without application to the manufacture of structural components for aerospace or automotive products. Broadening the applicability of 3D printing to obtain mechanically strong components for aerospace and automotive structures is a major goal of industrial fabrication.

Carbon-fiber composites show good mechanical performance because they incorporate the attributes of carbon fibers, which show high strength and stiffness, with those of polymer resins or metals. Fiber-reinforced thermoplastic (FRTP) composites can be applied in passenger automobiles to achieve high recyclability and load-bearing capability^13^. “Green” composites, based on natural fibers derived from plants and biodegradable resins, are in high demand to meet regulatory requirements of recyclability^14^,^15^,^16^. However, the conventional fabrication methods for composites require expensive facilities and equipment, such as autoclaves and complex rigid molds, hindering the wide application of composites.

The 3D printing of composite materials with enhanced mechanical properties is under extensive exploration^17^,^18^,^19^,^20^,^21^. Various reinforcements, such as carbon black^22^, reinforcing platelets^20^, and short fibers including chopped carbon fibers^23^, polymer fibrils^24^, carbon nanotubes^25^,^26^, and glass fibers^27^ have been used. In most such studies, the additives were mixed into thermoplastic filaments prior to being loaded into the printer, where the mixture replaced the conventional thermoplastic filament. However, some 3D printers use two printer heads for fabricating composites^28^. These composites usually show poor mechanical properties compared to composites manufactured by conventional methods, because composites reinforced with short fibers or particles are mechanically inferior to composites reinforced with continuous fibers^29^,^30^,^31^. Some 3D-printed composites are fabricated to achieve specific functionalities outside of mechanical behavior, such as high electric conductivities^22^,^26^, low coefficients of thermal expansion, and high thermal conductivities^32^.

To improve the mechanical properties of 3D-printed composites, a printer capable of fabricating continuous fiber-reinforced plastics is much in demand. One 3D printer using a plastic filament impregnated with carbon fibers has been developed^33^. However, in this process, the resin-fiber combination is predetermined; the selection of any other resin or reinforcing fibers for 3D printing is impossible. If the 3D printing of continuous FRTP of varied materials becomes possible, significant weight-saving can be achieved by optimizing the fiber direction in the printed components. In addition to applications in the automotive and aerospace industries, the technology would be well-suited to manufacture a wide variety of custom products in small quantities, such as load-bearing composite orthopedic implants^34^ and prosthetic limbs^35^,^36^ in the healthcare industry. Realizing the 3D printing of composites could supplant the conventional laborious manufacturing of composites, thus becoming the next-generation standard of composite fabrication (or “Composites 2.0”).

This study investigates the 3D printing of continuous FRTP, which combines fibers and resin in a nozzle based on FDM printing. We refer to composites obtained by direct digital manufacturing (DDM) as Composites 2.0 (as mentioned above), and those obtained by conventional manufacturing as Composites 1.0. A fiber-reinforced plastic obtained by Composites 2.0 technology would be a fully structurally optimized material, with the fiber direction and volume fraction precisely controlled at every location in the composite material. As proofs of concept, we have used straight tows of carbon fibers or twisted yarns of jute fibers as reinforcements in a matrix resin of polylactic acid (PLA). The carbon fiber composites demonstrate excellent mechanical properties, while the jute fiber composites are examples of plant-sourced composites^37^,^38^. The test specimens were printed along the longitudinal direction, parallel to the direction of fiber alignment. The mechanical properties of the 3D-printed continuous fiber composites were measured by tensile testing and compared with the performance of neat PLA resin.

## Results

### 3D printing of continuous fiber composites by in-nozzle impregnation

**Figure 1:**
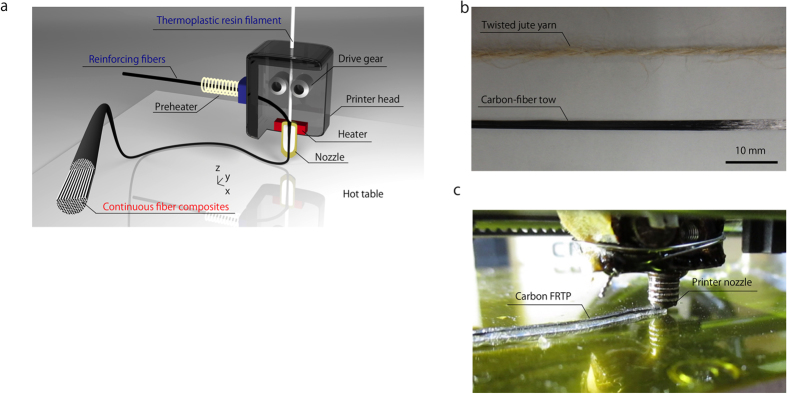
(**a**) Schematic of the 3D printer head used to produce continuous FRTPs using in-nozzle impregnation based on FDM. (**b**) Continuous fiber reinforcements used for 3D printing. (**c**) Photograph of the 3D printing of a CFRTP.

Figure 1(a) shows a schematic of the 3D printer head used for the production of continuous FRTP composites by in-nozzle impregnation. The thermoplastic resin filament and reinforcing fibers, i.e., the carbon fiber tow or twisted jute-fiber yarn (Fig. 1(b)), are separately supplied to the printer head. The reinforcing fibers are heated using a nichrome wire before entering the nozzle, to enhance the permeation of the fiber bundles with thermoplastic resin; the heat diffuses to the resin and decreases the viscosity of the PLA. The resin filament is transferred using drive gears and a stepping motor, while the reinforcing fibers are directly supplied to the nozzle. No additional devices are required for feeding the reinforcing fibers, because the fibers are automatically supplied to the head by the movement of the resin filament. The resin filament is melted by the heater inside the printer head, consolidating the reinforcing fibers and the resin in the heated section. The resin-reinforced fibers are extruded from the nozzle and laminated onto a hot table for the layer-by-layer fabrication of solid components. Fig. 1(c) shows the 3D printing process for manufacturing continuous carbon fiber-reinforced composites.

The 3D printer for obtaining continuous-fiber composites was developed by modifying the printer head of the commercially available FDM 3D printer Blade-1 printer with a preheating system. This modified printer was used to print carbon fiber-reinforced thermoplastics (CFRTPs), as shown in supplementary Fig. 1. A modified FlashForge printer was used to print jute fiber-reinforced thermoplastic (JFRTP) green composites (no preheating was used for the JFRTPs to prevent the degradation of the jute fibers). Unmodified PLA was also printed using the Blade-1 printer, as a comparison to measure the mechanical enhancement achieved by reinforcing the resin with continuous fibers. In the Blade-1 printer, the printer head moves along the *x* and *z* directions, whereas the hot table moves in the *y* direction to enable 3D fabrication. The diameter of the resin filament is 1.85 mm, exceeding the 1.4 mm diameter of the nozzle. Hence, the molten resin inside the nozzle is pushed out from the solid resin; the pressure facilitates the impregnation of molten resin into the fiber bundles in the nozzle. Since a large nozzle diameter was used in this pilot study to prevent clogging of the reinforcing fibers, the layer resolution was relatively low.

The PLA filament and carbon or jute fiber bundles were heated to 210 °C in the nozzle, while the temperature of the hot table was set to 80 °C. For CFRTPs and JFRTPs, the fiber/filament feeding speed and nozzle head speed were 100 mm/min and 60 mm/min, respectively. The difference in feeding speeds for the carbon and jute fibers prevented fiber stacking, which depended on the fiber type, within the nozzle. The fiber volume fraction (*v*_*f*_) of the FRTPs was determined by the supplied amounts of reinforcing fibers and an optical examination of the cross-section of the extruded filament, measured by scanning electron microscopy. The *v*_*f*_ of CFRTP and JFRTP was 6.6% and 6.1%, respectively. The jute fiber had a density of 1.46 g/cm^3^ 39.

**Figure 2:**
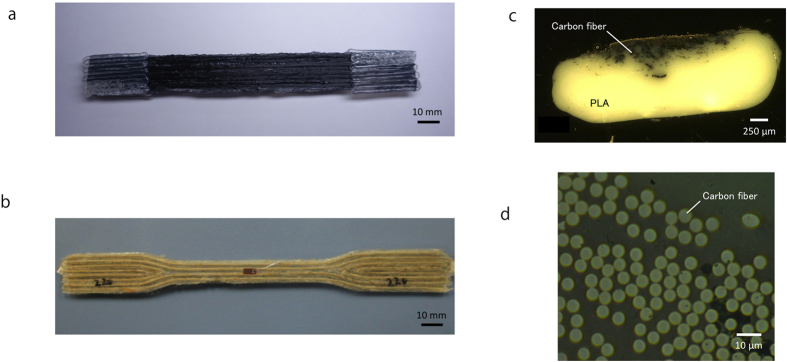
3D-printed (**a**) CFRTP and (**b**) dumbbell-shaped JFRTP tensile test specimens. (**c**) Cross-section and (**d**) magnified cross-section of the CFRTP specimen.

Figure 2(a,b) show the obtained printed CFRTP and JFRTP specimens. Figure 2 (c,d) show an overview and a magnification of a cross-section of a printed CFRTP part, confirming that the PLA resin has impregnated the fiber bundles. The cross-sectional areas of the printed composites are elliptical, despite the circular nozzle, because the filaments are compressed against the hot tables during printing.

### 3D printing of tensile test specimen

The continuous FRTP tensile test specimens were 3D-printed according to the JIS K 7162 standards^40^. For the CFRTPs, the test specimens were sliced from rectangular frames (see supplementary Fig. 2), while dumbbell-shaped JFRTP specimens were fabricated with a traversable CAD path. The ply created by this path was layered in the *z*-direction four times for a total specimen thickness of ~4 mm. The carbon or jute fibers were aligned along the longitudinal direction, which was chosen as the loading direction, and no fibers were aligned in the transverse (*y*) or thickness (*z*) directions in the gage region of the tensile test specimens. Therefore, the mechanical properties of the tensile specimen are orthotropic and maximized along the loading direction. Aligning the reinforcing fibers with the transverse and thickness directions would also be possible; however, the aspect of alignment is beyond the scope of this study and related experiments are not presented here. Neat PLA tensile specimens were also fabricated to demonstrate the reinforcing effect achieved by the continuous carbon or jute fibers. Four specimens each of PLA, CFRTP, and JFRTP were used for the tensile tests.

### Tensile tests

**Figure 3:**
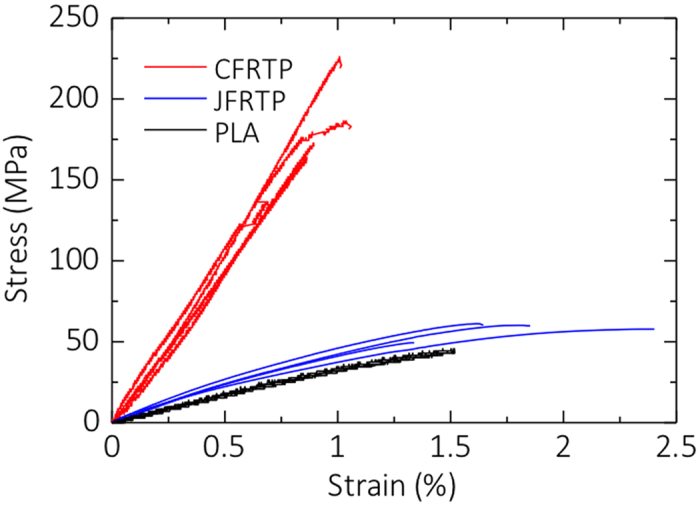
Stress-strain curves of PLA, unidirectional CFRTP, and unidirectional JFRTP specimens fabricated by 3D printing.

**Figure 4:**
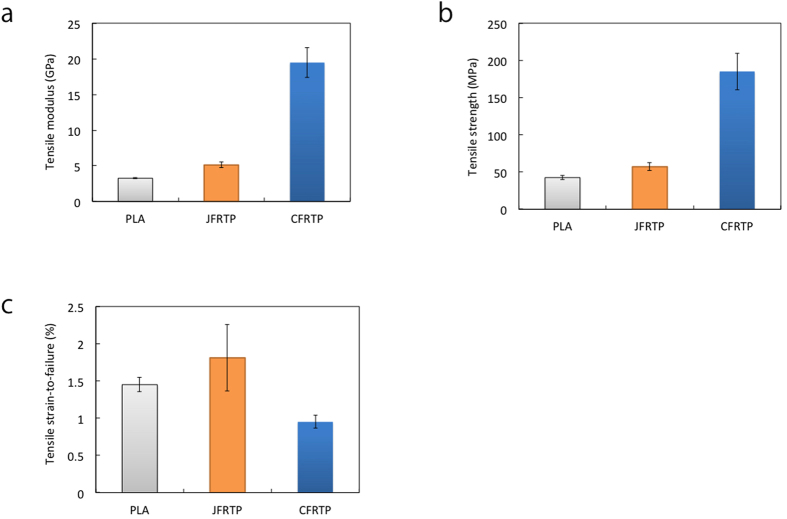
(**a**) Tensile modulus, (**b**) tensile strength, and (**c**) tensile strain-to-failure of specimens fabricated by 3D printing.

Figure 3 shows the stress-strain curves of PLA, CFRTP, and JFRTP. In the CFRTP specimen, the stress linearly increases before fracture occurs. A stress drop is observed in some specimens before failure. The stress-strain curve of JFRTP shows non-linearity; the tangential modulus decreases with increasing strain. No stress drop is observed before failure. The average values and standard deviation of the tensile modulus, tensile strength, and tensile strain-to-failure for the different specimens are shown in Fig. 4 (see supplementary Table 1 for a listing of the values). The tensile modulus and strength of 3D-printed CFRTP are 19.5 (±2.08) GPa and 185.2 (±24.6) MPa, respectively, which are 599% and 435% of those of the PLA specimen. Therefore, the mechanical properties are highly improved by 3D printing the PLA with continuous carbon fiber. The tensile strain-to-failure of CFRTP is decreased to 0.95 (±0.0873)% from the value of 1.45 (±0.0945)% shown by the PLA specimens, because of the low strain-to-failure characteristics of the carbon fibers. We also tested the samples by three-point bending; the flexural strength was 133 MPa, flexural modulus was 5.93 GPa, and flexural strain-to-failure was 4.09%. However, because the fibers were distributed non-uniformly in the cross-section of the specimen, these flexural properties should be understood as reference values.

The tensile strength of JFRTP is not improved significantly compared to that of CFRTP. For JFRTP, the tensile modulus and strength are 5.11 (±0.41) GPa and 57.1 (±5.33) MPa, corresponding to 157% and 134% of those shown by the PLA specimen, respectively. The tensile modulus of JFRTP is obtained from the tensile strain in the range of 0.05% to 0.25%, based on the JIS K 7162 standard, although the tangential modulus decreases with increased stress above this strain range. The decrease in tangential modulus and the lack of improvement in the tensile strength can be attributed to the degradation of fiber-resin interactions with increased applied stress.

While the mechanical property and volume fraction of the fiber reinforcement affected the properties of the FRPT, the composite properties are also affected by the configuration of the twisted yarn reinforcement^41^,^42^,^43^. In the 3D printing method employed, no tension was applied to the jute fiber when the yarn was fed to the nozzle. This led to a non-uniform configuration of the twisted jute yarn fibers, which may have created weak points in the printed filament. When using a twisted yarn as reinforcement, an appropriate tension and torsional moment must be applied to the yarn during 3D printing, which would assist the uniform molding of the FRTP.

### Fracture imaging

**Figure 5:**
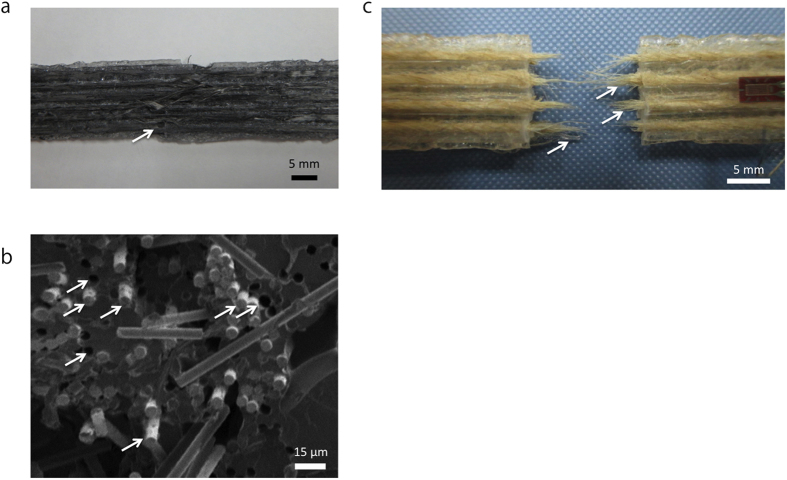
Fracture of a unidirectional continuous FRTP fabricated by 3D printing. Fiber pullout due to tensile fracture observed as an (**a**) overview and (**b**) scanning electron microscopy image of the CFRTP specimen. (**c**) Fiber pullout in the JFRTP specimen.

Figure 5(a,b) show the fracture surface and cross-sections, respectively, obtained by scanning electron microscopy (SEM) after the tensile testing of the CFRTP specimen, while Fig. 5 (c) shows the fracture surface of the JFRTP specimen. For comparison, an image of the fracture surface of the PLA specimen is shown in the supplementary Fig. 3. In both the CFRTP and JFRTP specimens, fiber pullout attributed to fracture is observed both macroscopically and microscopically. This indicates that the adhesion between the fibers and the thermoplastic resin is insufficient; hence, treatment of the fiber surfaces is required to achieve further enhancement in mechanical properties. Poor adhesion between thermoplastic resin and reinforcing fibers is common in the development of FRTPs. Voids are observed between filaments in the CFRTP sample because the molten CFRTP filament, once ejected from the nozzle, becomes laminated and assumes an elliptical shape. Voids between filaments are also common in FDM-type 3D printing^44^,^45^. In the future, similar candidate 3D printed composites should be modeled by numerical simulation to predict mechanical strength, but the fiber bundle distribution and void existence may require special consideration.

### Comparison with conventional polymer-based 3D printing

**Figure 6:**
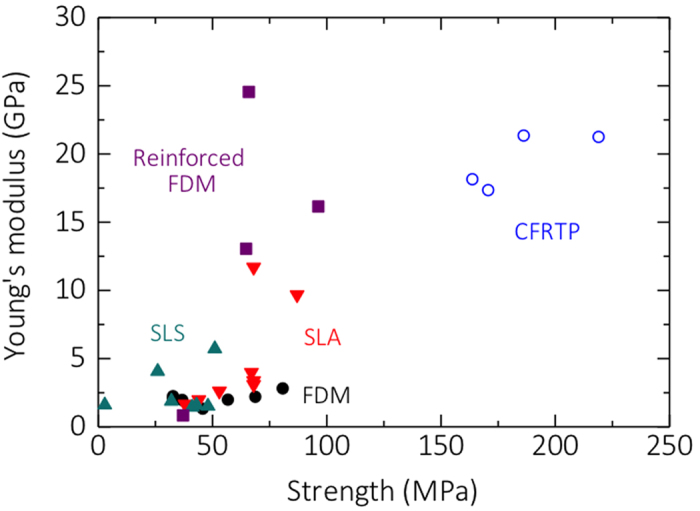
Young’s moduli and strengths of continuous carbon-fiber composites fabricated in the present study compared with composites fabricated by FDM^21^,^23^,^25^ and using commercially available 3D printers, such as SLS^48^, SLA^48^, and FDM^49^.

Figure 6 shows the Young’s modulus and strength values obtained in the present study (indicated as continuous-fiber composites) and the values shown by conventional polymer-based 3D-printed samples reported in the literature, including samples fabricated using commercially available 3D printers and those reinforced by FDM using nano-clay platelets^21^, short carbon fibers^23^, and carbon nanotubes^25^. The continuous carbon-fiber composites fabricated in the present study show superior Young’s moduli and strengths compared with materials fabricated using commercially available 3D printers, whether these are operated by selective laser sintering, stereolithography, or FDM. Further, the Young’s moduli of composites from this study exceed those shown by composites obtained by FDM, with the exception of composites fabricated by FDM using nanoclay platelets as reinforcements^21^, because in this specific case, the resin used was epoxy, which exhibits high mechanical properties. As the fiber volume fraction of the printed composites in the present study is low and the Young’s modulus can be improved by increasing the fiber volume fraction, the performance of our composites could be improved. The carbon composites used in aerospace structures typically have fiber volume fractions of ~67%. We estimate the upper limit of fiber volume fraction in FDM 3D printing to be ~40–50%, because of the necessity of running fibers through the printer nozzle. The strength of the composites from this study was approximately twice that of the composites fabricated by FDM. Hence, the process we propose to fabricate continuous fiber composites expands the applicability of 3D printing to the manufacture of load-bearing components, which cannot be realized by conventional 3D printing.

## Methods

### Materials

Commercially available PLA filament (Hotproceed, 1.75 mm diameter) was used as the matrix material. Polyacrylonitrile (PAN)-based carbon fibers (T800S-10E, Toray) and twisted jute natural plant fibers^46^ were used as continuous reinforcements. For the carbon fibers, several thousand filaments were extracted from the straight 24000 filaments and supplied to the printer head. The carbon fibers were used as-is with epoxy sizing. The mechanical properties of the carbon fibers and jute fibers are shown in supplementary Table 1^47^. For the jute fibers, a single strand of yarn was extracted from the twisted 500-Tex double yarn. No surface treatment was performed after purchasing.

### 3D printers

The FDM Blade-1 3D printer, produced by Hotproceed, Japan, was modified for printing CFRTP, while the FlashForge printer, produced by Zhejiang FlashForge 3D Technology, China, was modified for printing JFRTP.

### Tensile tests

Quasistatic tensile tests were performed on the CFRTP, JFRTP, and PLA specimens using a universal tester (AG-IS 150 kN, Shimadzu) at a crosshead speed of 1.0 mm/min. A strain gage was attached to the specimen surface to measure strain. The specimen configuration was rectangular with PLA end tabs for the CFRTP, while the JFRTP and PLA specimens were dumbbell-shaped without end tabs. The shoulder portions of the specimen were clamped directly by the wedge grips of the tester and a tensile load was applied uniaxially to the specimen. We tested four specimens each of CFRTP, JFRTP, and PLA.

## Additional Information

**How to cite this article**: Matsuzaki, R. *et al.* Three-dimensional printing of continuous-fiber composites by in-nozzle impregnation. *Sci. Rep.* **6**, 23058; doi: 10.1038/srep23058 (2016).

## Supplementary Material

Supplementary Information
