# Supplementary material for: Three-dimensional printing of continuous-fiber composites by in-nozzle impregnation
Source: Sci Rep. 2016 Mar 11;6:23058. doi: 10.1038/srep23058 (PMC4786850; doi:10.1038/srep23058)
Supplement: Supplementary Information [file srep23058-s1.pdf]

Three-dimensional printing of continuous-fiber composites by in-nozzle impregnation

Ryosuke Matsuzaki[1,*], Masahito Ueda[2,*], Masaki Namiki[2], Tae-Kun Jeong[2], Hirosuke Asahara[2], Keisuke Horiguchi[1], Taishi Nakamura[1], Akira Todoroki[3], Yoshiyasu Hirano[4]

[1] Tokyo University of Science, 2641 Yamazaki, Noda, Chiba 278-8510, Japan
[2] Nihon University, 1-8-14 Kanda-surugadai, Chiyoda, Tokyo 101-8308, Japan
[3] Tokyo Institute of Technology, 2-12-1 O-okayama, Meguro, Tokyo 152-8551, Japan
[4] Japan Aerospace Exploration Agency, 6-13-1 Osawa, Mitaka, Tokyo 181-0015, Japan

[*] These authors contributed equally to this work.

Correspondence and requests for materials should be addressed to R.M. (email: rmatsuza@rs.tus.ac.jp) or to M.U. (ueda@mech.cst.nihon-u.ac.jp) or to A.T. (email: atodorok@ginza.mes.titech.ac.jp).

Supplementary Information

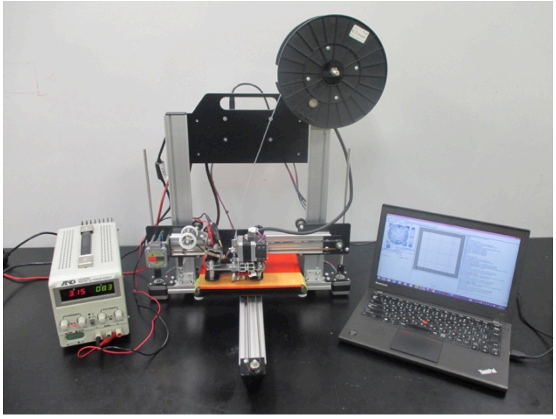

Fig. 1 The 3D printer used for fabricating continuous-fiber composites by in-nozzle impregnation.

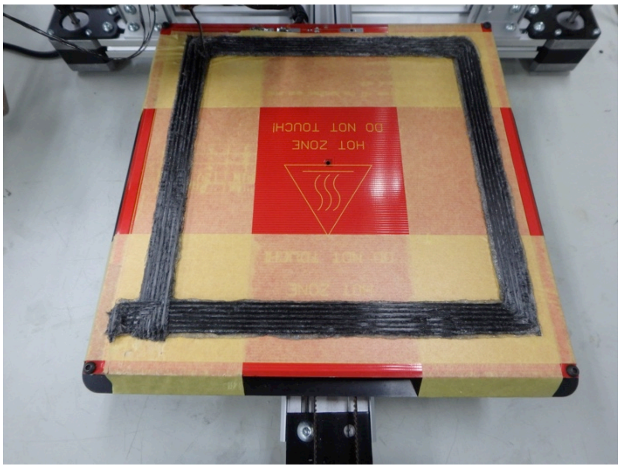

Fig. 2 Specimen fabrication of CFRTPs by 3D printing. Four specimens were sliced from the four sides.

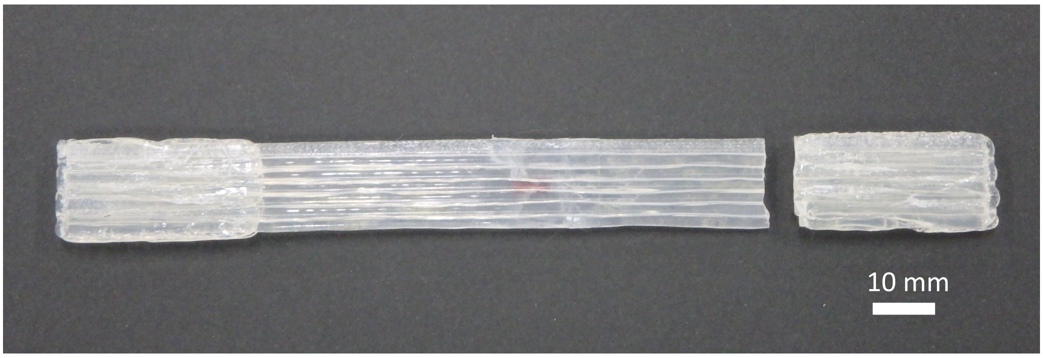

Fig. 3 PLA specimen fabricated by 3D printing after the tensile test.

Table 1. Tensile mechanical properties of materials used for 3D printing and 3D printed specimens.

	Carbon fiber[47]	Jute fiber	3D printed specimen<br>PLA	3D printed specimen<br>CFRTP (v_f = 0.066)	3D printed specimen<br>JFRTP (v_f = 0.061)
Tensile modulus [GPa]	294	27.4 (±7.81)	3.25 (±0.0577)	19.5 (±2.08)	5.11 (±0.410)
Tensile strength [MPa]	5880	417 (±111)	42.6 (± 2.62)	185.2 (± 24.6)	57.1 (±5.33)
Tensile strain-to-failure [%]	2.0	2.81 (±0.324)	1.45 (±0.0945)	0.95 (±0.0873)	1.81 (±0.448)
